# Comparison between carrier transport property and crystal quality of TlBr semiconductors

**DOI:** 10.1038/s41598-024-76005-9

**Published:** 2024-10-24

**Authors:** Kenichi Watanabe, Yusuke Sugai, Sota Hasegawa, Seishiro Tanaka, Keitaro Hitomi, Mitsuhiro Nogami, Takenao Shinohara, Yuhua Su, Joseph Don Parker, Winfried Kockelmann

**Affiliations:** 1 Department of Applied Quantum Physics and Nuclear Engineering, Kyushu University, 744 Motooka, Nishi-ku, Fukuoka, 819-0395 Japan; 2 Department of Quantum Science and Energy Engineering, Tohoku University, Aoba, Sendai, Aoba-ku, Aramaki 980-8579 Japan; 3 J-PARC Center, Japan Atomic Energy Agency, 2-4 Shirakata, Tokai-Mura, Ibaraki 319-1195 Japan; 4 Neutron Science and Technology Center, Comprehensive Research Organization for Science and Society, 162-1 Shirakata, Tokai-Mura, Ibaraki 319-1106 Japan; 5 ISIS Facility, Rutherford Appleton Laboratory, Science and Technology Facilities Council, Harwell, Oxfordshire OX11 0QX UK

**Keywords:** Nuclear physics, Techniques and instrumentation, Materials for devices, Electrical and electronic engineering

## Abstract

Thallium bromide (TlBr) semiconductor detectors are being developed as promising candidates for high-detection-efficiency, high-energy-resolution, and room-temperature gamma-ray spectrometers. This study presents methods for evaluating TlBr crystal quality and carrier transport characteristics using neutron Bragg-dip imaging and the time-of-flight method for pulsed-laser-induced carriers, respectively. Neutron Bragg-dip imaging effectively determines the crystal orientation distribution, revealing crystal imperfections and grain boundaries. Time-of-flight measurements provide a spatial distribution of carrier mobility. In this study, two samples obtained from both the upstream and downstream region in the crystal ingot were evaluated. Although both samples show similar crystal quality, the upstream sample showed high carrier mobility across all areas, whereas the downstream sample exhibits low mobility in some areas. These findings suggest that, at least within the range of carrier mobility currently obtained, the effect of crystal integrity on carrier mobility is less significant than that of impurities. In conclusion, combining neutron Bragg-dip imaging with carrier mobility measurements offers a comprehensive approach to evaluating and improving TlBr detectors.

## Introduction

Highly efficient gamma-ray detectors require a bulky crystal to effectively capture high-energy gamma rays. Gamma-ray detectors are mainly categorized into two types: scintillators and semiconductors. Although scintillator-based concept allows for relatively easy fabrication of large detectors, they have a limitation in energy resolution. On the other hand, semiconductor-based detectors show relatively high energy resolution. However, the fabrication process to realize large detectors has not been established, except for high-purity germanium (HPGe) detectors. This is the reason why the HPGe detector is the gold standard gamma-ray detector. However, HPGe detectors have a disadvantage in that they require cooling. Therefore, many researchers are developing alternatives to the HPGe system, such as CdTe, CdZnTe detectors, which can operate at room temperature.

One of the promising candidate materials for semiconductor detectors is thallium bromide (TlBr)^1–10^. Since TlBr has a wide bandgap energy of 2.68 eV, TlBr detectors can operate at room temperature. In addition, since this material consists of high atomic number elements (Tl = 81, Br = 35) and has high density of 7.56 g/cm^3^, it is expected to show high detection efficiency. Owing to improvements in the purification process, the carrier transport characteristics of TlBr has been improved^11^. Consequently, TlBr semiconductor detectors have a relatively high mobility-lifetime product of the order of 10^−3^ cm^2^/V and show high energy resolution of 1% for 662 keV gamma rays. The carrier transport characteristics in semiconductor materials is considered to depend on both impurity and crystal quality. Owing to the purification process, the impurity level in TlBr crystals is well suppressed to be quite low. To realize large-volume and high-quality detectors, quality evaluation methods of the TlBr crystal should be established.

So far, we have developed the evaluation method of crystal orientation distribution of TlBr based on neutron Bragg-dip analysis, which is a novel neutron diffraction imaging method^12,13^. The validity of this method has been confirmed through comparison with electron backscatter diffraction (EBSD) analysis^13,14^. In addition, we developed the evaluation method of spatial distribution of carrier transport characteristics based on the time-of-flight method for pulsed-laser-induced carriers^15,16^. In this paper, we discuss the relationship between the crystal orientation distribution and carrier transport characteristics.

## Methods

### Sample preparation

**Fig. 1:**
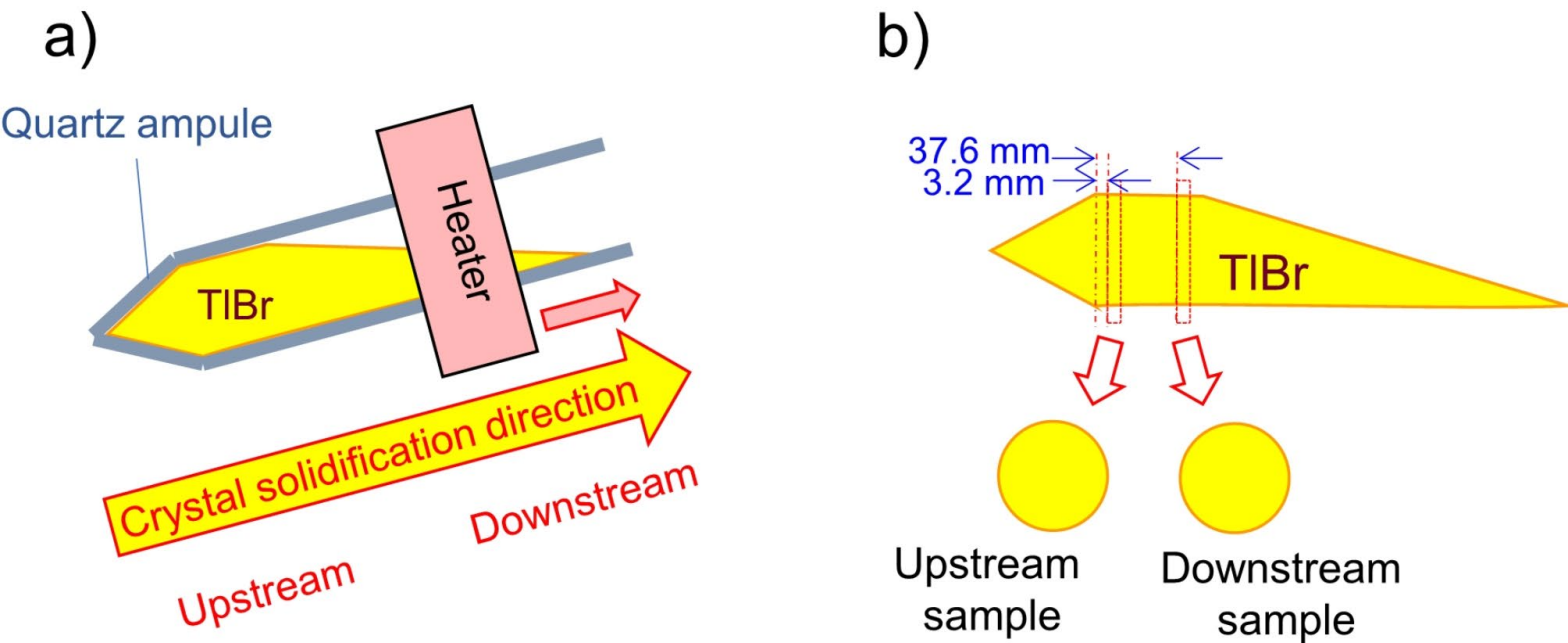
Schematic drawing of (**a**) the tilted horizontal traveling zone method and (**b**) cutting positions of disk wafers from a crystal ingot.

We prepared two disk-shaped TlBr crystal wafers. A commercially available TlBr powder with 99.999% purity was used as the starting material. The powder was loaded and sealed into a quartz ampule. The raw material was purified by multi-pass zone refining^11^. Then, a TlBr crystal was grown by the tilted-horizontal traveling zone method, as shown in Fig. 1a. The grown TlBr crystal ingot was cut into disks perpendicular to the crystal solidification direction. Two disks were cut from different positions of the ingot, as shown in Fig. 1b. They are called the upstream and downstream samples. The thicknesses of the disk wafers were 2.88 mm and 2.81 mm for the upstream and downstream samples, respectively. After the cutting process, the wide surfaces on both sides of the disks were polished. The disk wafers were used for the neutron Bragg-dip measurements. The wafers were also studied by EBSD for a cross-verify the crystal orientation distribution.

After the diffraction measurements, gold electrodes were formed on the both surfaces of the disks using a vacuum evaporation method. The thickness of the electrodes was approximately 100 nm. Carrier mobility measurements were performed on the TlBr crystals with gold electrodes.

### Neutron Bragg-dip imaging

**Fig. 2:**
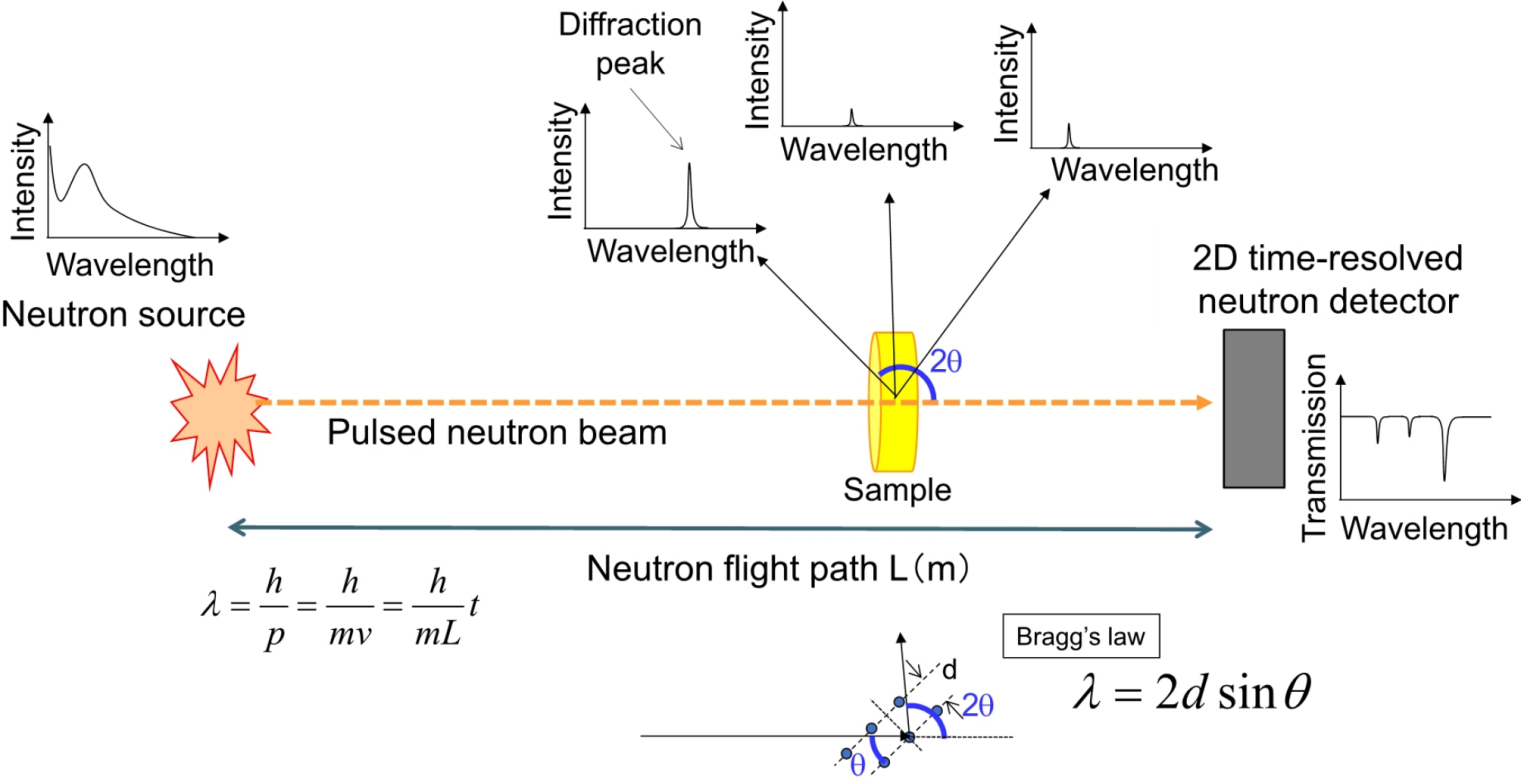
Conceptual drawing of neutron Bragg-dip imaging.

**Fig. 3:**
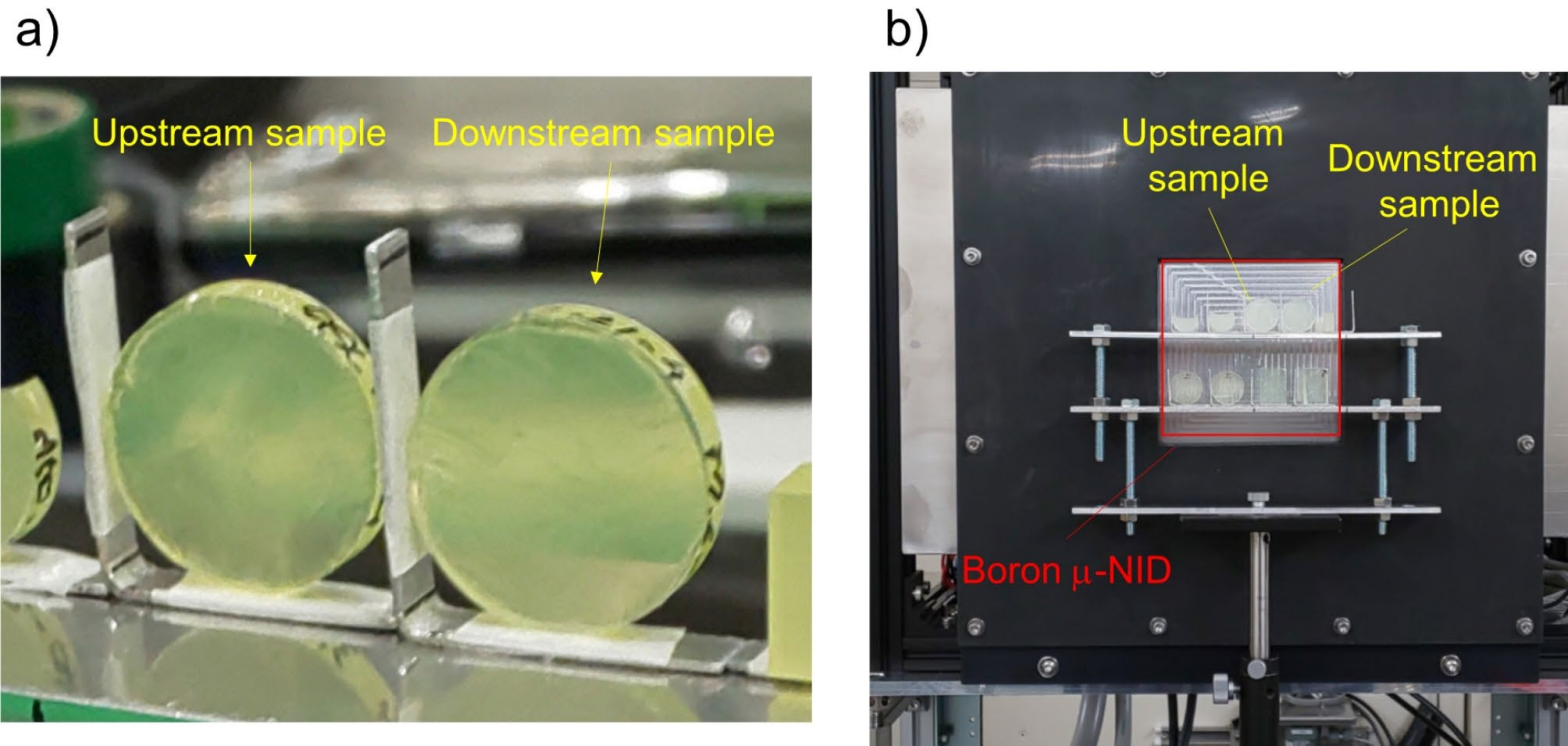
Photographs of (**a**) the upstream and downstream samples and (**b**) samples placed in front of the boron µ-NID detector.

We conducted a neutron Bragg-dip imaging experiment to determine the TlBr crystal orientation distribution^12,13^. Neutron Bragg-dip imaging is based on a neutron diffraction technique using energy-resolved neutron transmission. Figure 2 shows a conceptual drawing of the neutron Bragg-dip imaging setup. We acquired energy-resolved images of TlBr crystals at RADEN beamline (BL-22) at the Material and Life Science Facility (MLF) in the Japan Proton Accelerator Research Complex (J-PARC)^17^. Figure 3 shows a photograph of the TlBr samples measured at the RADEN beamline. At the pulsed neutron facility, we can obtain information about the neutron energy or wavelength by using the time-of-flight technique. We employed a two-dimensional time-resolving neutron detector, a micro pixel chamber-based detector with a boron neutron converter (boron µ-NID)^18^. Using the boron µ-NID, we can acquire the neutron transmission spectra at all two-dimensional pixels. The transmission spectrum shows some dip structures at wavelengths for which neutrons are diffracted from the incident neutron beam. The pattern of these dip positions and intensities depends on the crystal structure, crystal orientation, and is also affected by extinction.

We applied the pattern matching technique to estimate the crystal orientation from the experimental dip pattern. From information of the crystal structure^19^, such as the space group and the lattice parameters, and nuclear properties^20,21^, such as the neutron scattering length and the cross section, we can estimate the diffraction intensities and d-spacings of lattice planes corresponding to each Miller index by using the nxs code, which was developed by M. Boin^22^. We note that diffraction intensities simulated by the nxs code do not take the extinction effect into account which needs to be considered for large crystallites^23,24^. The space group of TlBr is Pm-3 m (#221) and the lattice parameter is *a* = 0.398 nm^19^, from which one can calculate d-spacings and diffraction intensities for a multitude of crystal places.

**Fig. 4:**
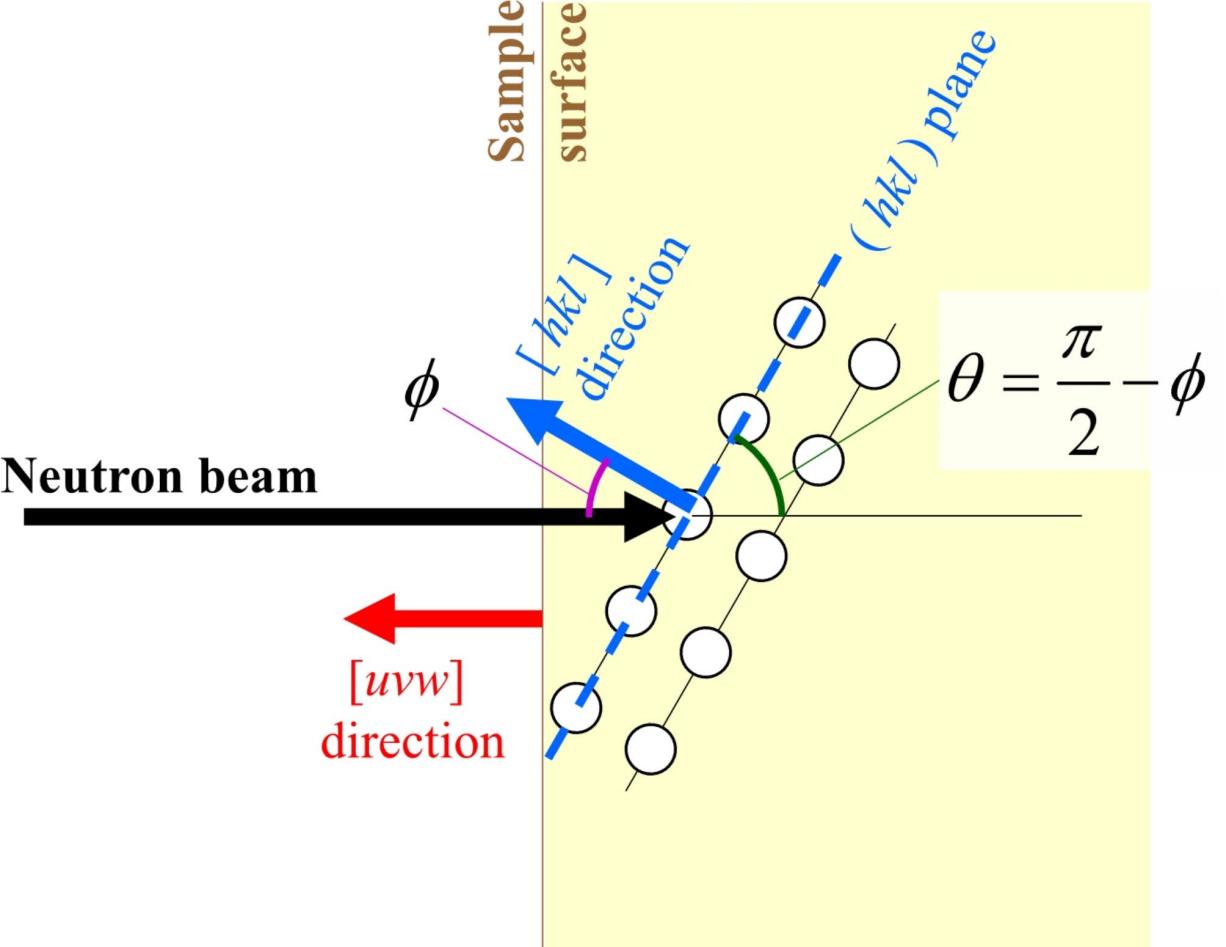
The geometrical relation between the neutron beam direction, Miller index vector of the sample surface, and a given crystal-lattice plane (hkl).

In the experiments, the neutron beam was incident perpendicularly on the sample surface. The neutron transmission spectra reflect the crystal orientation relative to the neutron beam direction. To uniquely define the crystal orientation, two vectors are required. However, with a single transmission measurement only one orientation vector can be determined, leaving the crystal orientation around the neutron beam axis undefined^23,24^. In this study, determining the complete crystal direction was not essential. Rather it was sufficient to assess the degree of alignment of the crystal orientation, which represents the crystal quality. We used the Miller index vector [*uvw*] of the sample surface plane to represent the sample orientation. The Miller index vector [*hkl*] represents the normal vector to the crystal-lattice plane defined by the Miller index of (hkl). Therefore, the Miller index for the sample surface is (*uvw*). Figure 4 shows the geometrical relation between the neutron beam direction, Miller index vector of the sample surface, and a given crystal-lattice plane (*hkl*). Here, an angle *φ* between the normal vectors of the sample surface (*uvw*) and the (*hkl*) plane is calculated by^25^.

1 $$\phi ={\cos ^{ - 1}}\left( {\frac{{hu+kv+lw}}{{\sqrt {{h^2}+{k^2}+{l^2}} \,\,\sqrt {{u^2}+{v^2}+{w^2}} }}} \right)$$

In this case, the diffraction angle *θ* is written by

2 $$\theta =\frac{\pi }{2} - \phi$$

Therefore, the neutron wavelength *λ* corresponding to the diffraction from the (*hkl*) plane is written by

3 $$\lambda =2{d_{hkl}}\sin \theta$$

where *d*_*hkl*_ is the d-spacing of the (*hkl*) plane. In this way, once the crystal orientation, i.e. the Miller index for the sample surface is set, we can calculate the Bragg-dip wavelengths or positions. To define the Bragg-dip pattern, the positions of the dips are the most important parameters, but the intensities and widths are also required. Although Bragg-dip widths contain various information, such as crystal mosaicity, they are assumed to be constant for simplicity in this study. While diffraction intensities and corresponding Bragg-dip intensities were simulated by using the nxs code^22^, intensities do not take the extinction into account. The extinction effect is considered to be essential for large single crystallites, while, for our samples, crystal integrity was not particularly high and the extinction was not crucial in our case. Hence, for a given crystal orientation, we can calculate the Bragg-dip pattern. Further, dip-patterns for all possible crystal orientations were calculated as references for the pattern matching method.

As an index for the pattern matching degree, we employed a cosine similarity. First, the observed neutron transmission spectrum was converted to attenuation according to the following relation between the attenuation *A* and transmission *T*:

4 $$A= - \ln T$$

The experimental attenuation spectrum was regarded as a multi-dimensional vector and the similarity between it and the prepared dip patterns was calculated. The similarity *S* is defined as follows:

5 $$S=\cos \Theta =\frac{{\vec {p} \cdot \vec {e}}}{{\,\,\left| {\vec {p}} \right|\left| {\vec {e}} \right|\,\,}}$$

where $\vec {p}$and $\vec {e}$ are the prepared and experimental attenuation spectrum vectors, respectively. The cosine similarity indicates a different angle Θ between two spectrum vectors. A similarity closer to unity indicates a higher degree of matching between the two vectors. The cosine similarity is sensitive to differences in the positions of large value elements, that is, the dip positions, within a vector where most elements have small values. In such pattern matching method, the residual sum of squares, commonly used in least squares methods, is usually employed as the index. However, since the prepared vectors do not take the extinction effect into account, the cosine similarity is adopted as an index sensitive to positions rather than peak intensities. By finding the maximum similarity, we can estimate the crystal orientation with respect to the neutron beam.

For validation purposes, we also conducted EBSD measurements using a scanning electron microscope (SEM, Zeiss, ULTRA55) at the Ultramicroscopy Research Center, Kyushu University. Crystal orientation maps were created by analyzing the diffracted electron patterns^14^.

### Mobility measurement based on time-of-flight method for pulsed-laser-induced carriers

**Fig. 5:**
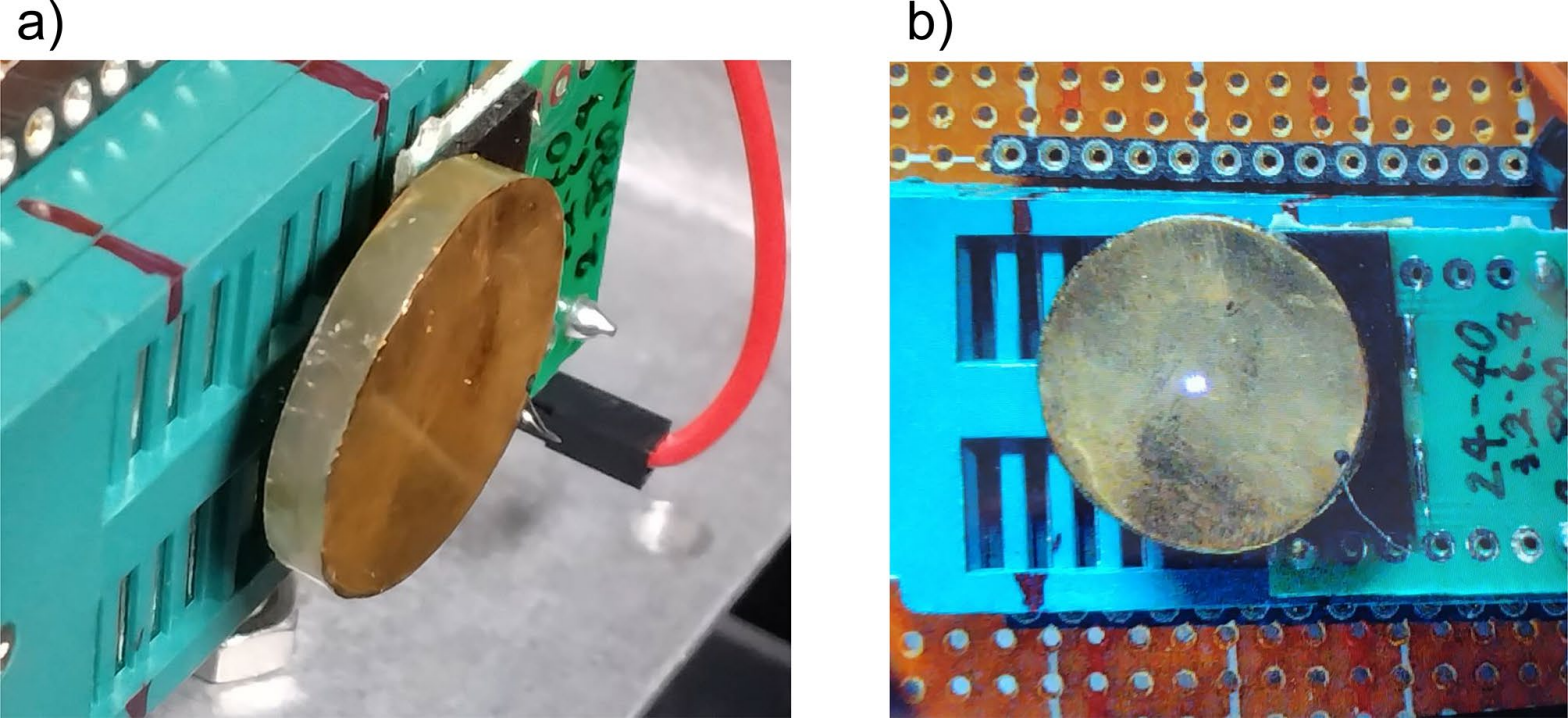
Photographs of (**a**) the TlBr planar detector with gold electrodes and (**b**) the detector irradiated with the pulsed laser.

The mobility measurement system consists of a pulsed laser, laser beam delivery optics, detector, signal amplification modules, and signal data acquisition system^16^. As the laser source, a pulsed laser (NPL41C, Thorlabs) with a wavelength of 405 nm was used. The pulse width was 128 ns. Although the maximum laser energy was 128 nJ, it was adjusted according to the experimental conditions. The laser beam was delivered and focused to the detector surface by using mirrors and a lens, creating a spot size of less than 0.5 mm. The laser beam was directed at right angle onto the gold electrode. Although the pulsed laser irradiated the gold electrode on the TlBr crystal, a small fraction of the laser light transmitted through a gold electrode and generating electron-hole pairs in the TlBr crystal. Preliminary measurements estimated the laser transmittance of a 100-nm-thick gold electrode to be approximately 10^−4^. The TlBr detector sample was mounted on a two-axis translation stage to enable scanning of the laser irradiation position on the sample. Figure 5 shows photographs of the detector sample irradiated with the pulsed laser. Due to the short attenuation length of 405 nm photons in TlBr, a significant number of electron-hole pairs were generated just below the gold electrode. By applying a negative voltage to the laser-irradiated gold electrode, generated electrons were driven to the opposite electrode, while holes were immediately collected at the laser-irradiated surface. A signal current was induced in the electrodes as the electrons moved. The induced current on the opposite electrode was amplified by a transimpedance amplifier (TIA60, Thorlabs) and digitized by an analog-to-digital converter (ADC, model 1820, CLEAE-PULSE). The pulsed laser and ADC were synchronized with 100 Hz trigger pulses. Data were collected and analyzed on a personal computer. The current pulse signals were averaged for 1,000 pulses to reduce noise contribution. One can determine the carrier velocity from the width of an induced current pulse and the detector thickness. We determined the carrier mobility by measuring the carrier velocity for several applied voltages.

## Results and discussion

### Neutron Bragg-dip imaging

**Fig. 6:**
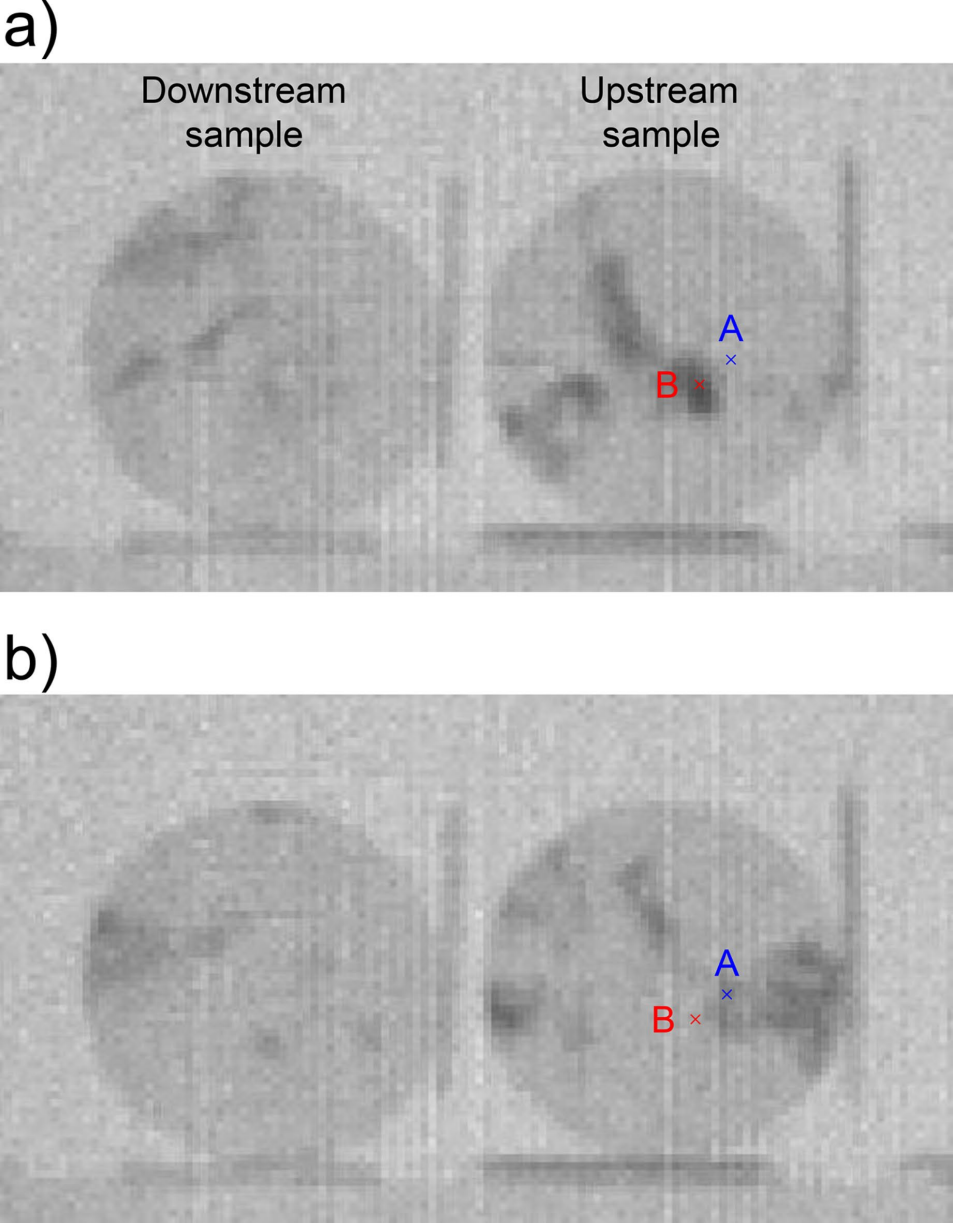
Neutron transmission images at neutron wavelengths of (**a**) 0.4698 nm and (**b**) 0.4775 nm.

**Fig. 7:**
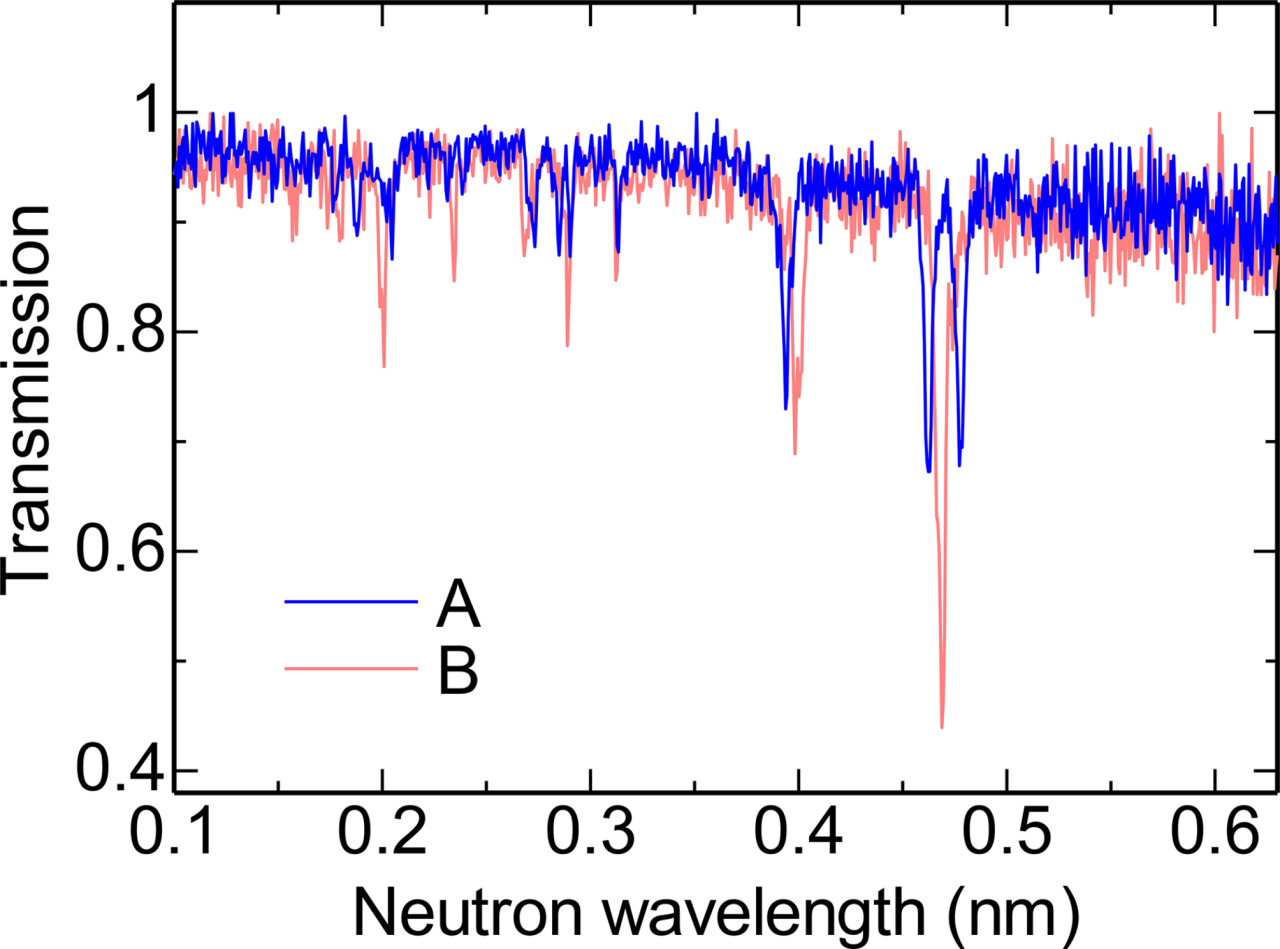
Neutron transmission spectra obtained at positions of A and B shown in Fig. 6.

Figure 6 shows the neutron transmission images for two slightly different neutron wavelengths. In these images, dark color indicates low neutron transmission. In dark regions, neutrons are diffracted away from the detector. As the neutron wavelength slightly changed, the dark regions shifted. This indicates that a crystal is imperfect and is slightly distorted. Figure 7 shows the neutron transmission spectra obtained at positions A and B, as marked in Fig. 6. Both positions display dip patterns that are similar in some aspects but slightly different in others. This indicates that each position has slightly different crystal orientations. Thus, the dip pattern provides information on the crystal orientation.

**Fig. 8:**
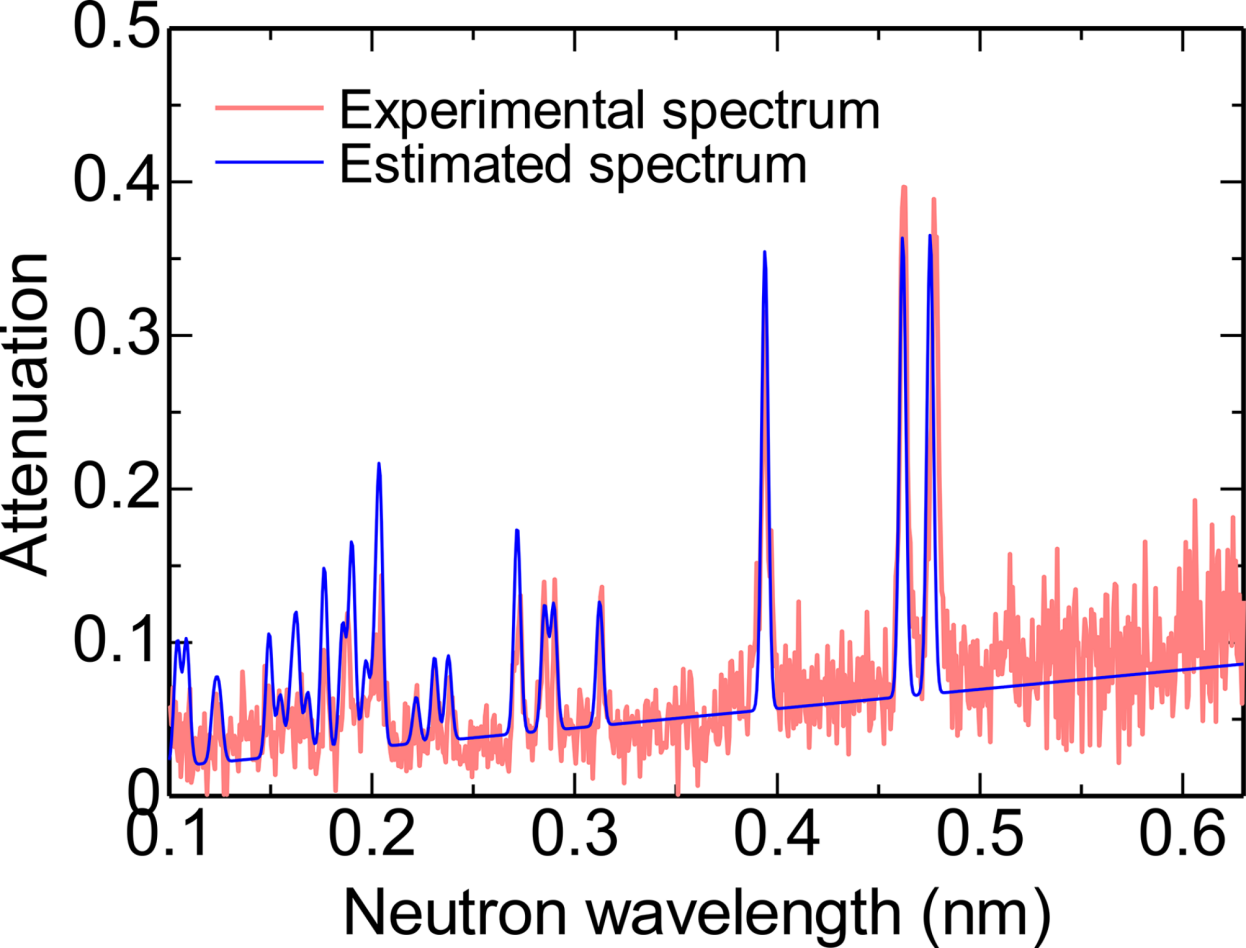
Neutron attenuation spectrum analysis by the pattern matching method for position A shown in Fig. 6. The estimated crystal orientation was [0.75 0.70 1.00]. The value of the similarity was 0.80.

Figure 8 shows an example of the attenuation spectrum estimated by the pattern matching method. Since the estimated spectrum closely matches the experimental one, the crystal orientation is considered to be well determined.

**Fig. 9:**
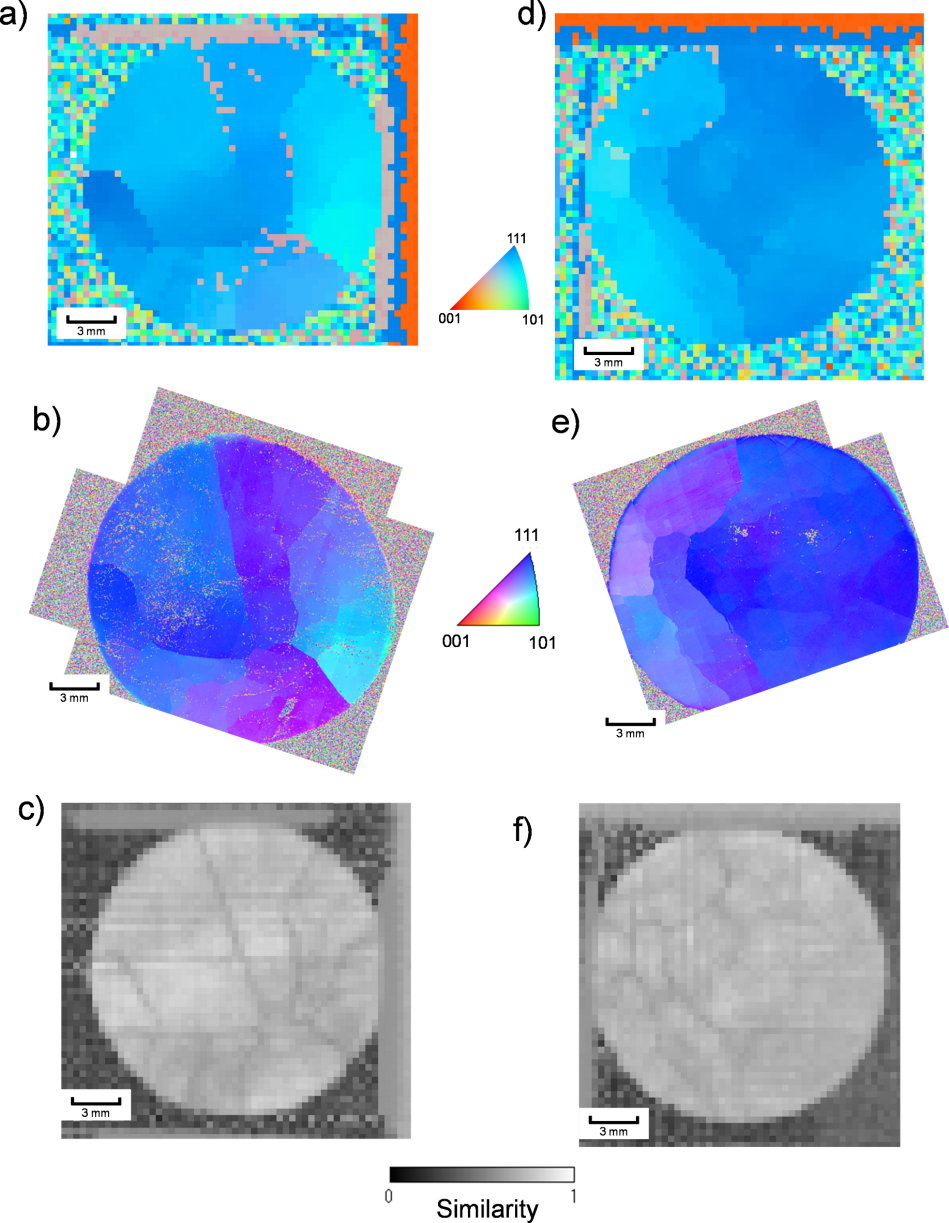
Crystal orientation maps of the TlBr wafers. (**a**–**c**) show the upstream sample. (**d**–**f**) show the downstream sample. The maps of (**a**, **d**) display the crystal orientation estimated by neutron Bragg-dip imaging. The maps of (**b**,**e**) show the crystal orientation determined by EBSD. The maps of (**c**,**f**) display the similarity criterion obtained in the neutron Bragg-dip analysis.

Figure 9 shows the crystal orientation maps of the TlBr disk wafers estimated by neutron Bragg-dip imaging. The crystal orientation maps determined by the EBSD analysis are also displayed. A comparison between neutron Bragg-dip and EBSD maps shows that both methods exhibit similar trends. While neutron Bragg-dip imaging provides information along the neutron beam penetrating the sample, EBSD only provides the information at the sample surface. Therefore, although there are slight differences between images created by the two methods, neutron Bragg-dip imaging is considered to correctly estimate the crystal orientations. This feature also has confirmed in the previous study^13^. Since both samples exhibit some crystal grain boundaries, they are not ideal single crystals.

**Fig. 10:**
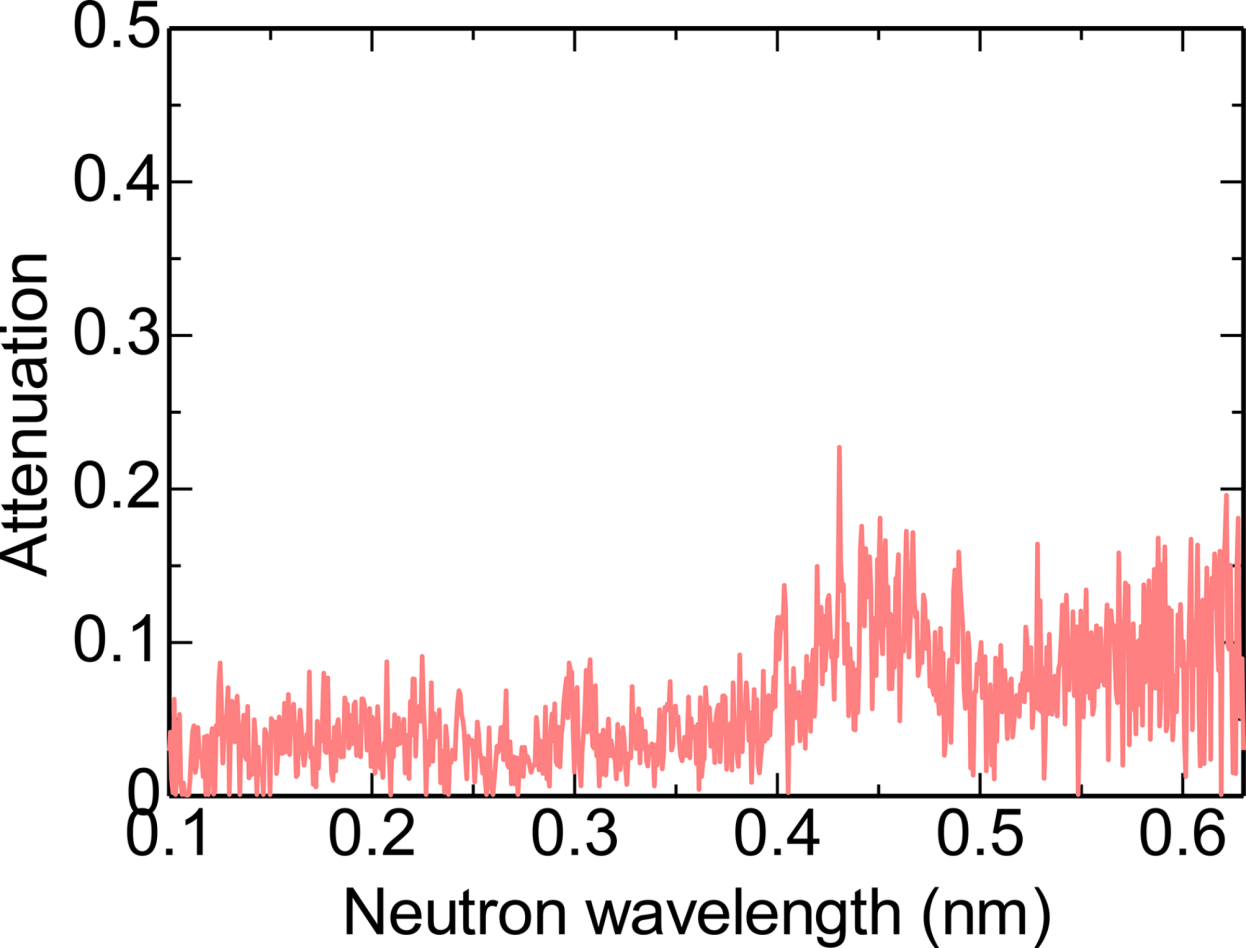
Neutron attenuation spectrum at the position having a low similarity value of 0.63.

In addition to the crystal orientation, maps of the similarity criterion are also shown in Fig. 9. With increasing mismatch of experimental and calculated dip patterns, the similarity value decreases. Figure 10 shows an example of the neutron attenuation spectrum obtained at the position having a low similarity value. At this position, no clear peaks can be observed. This means that the crystallites are not aligned along the neutron beam. In the black regions shown in Fig. 9c,f, the crystal orientations are misaligned along the thickness direction of the sample. Black regions are assumed to correspond to crystal grain boundaries.

Since both the upstream and downstream samples have some grain boundaries, the crystal is considered to have started growing from various positions. This suggests that there is room for improvement in the crystal growth process.

### Mobility measurement based on time-of-flight method for pulsed-laser-induced carriers

**Fig. 11:**
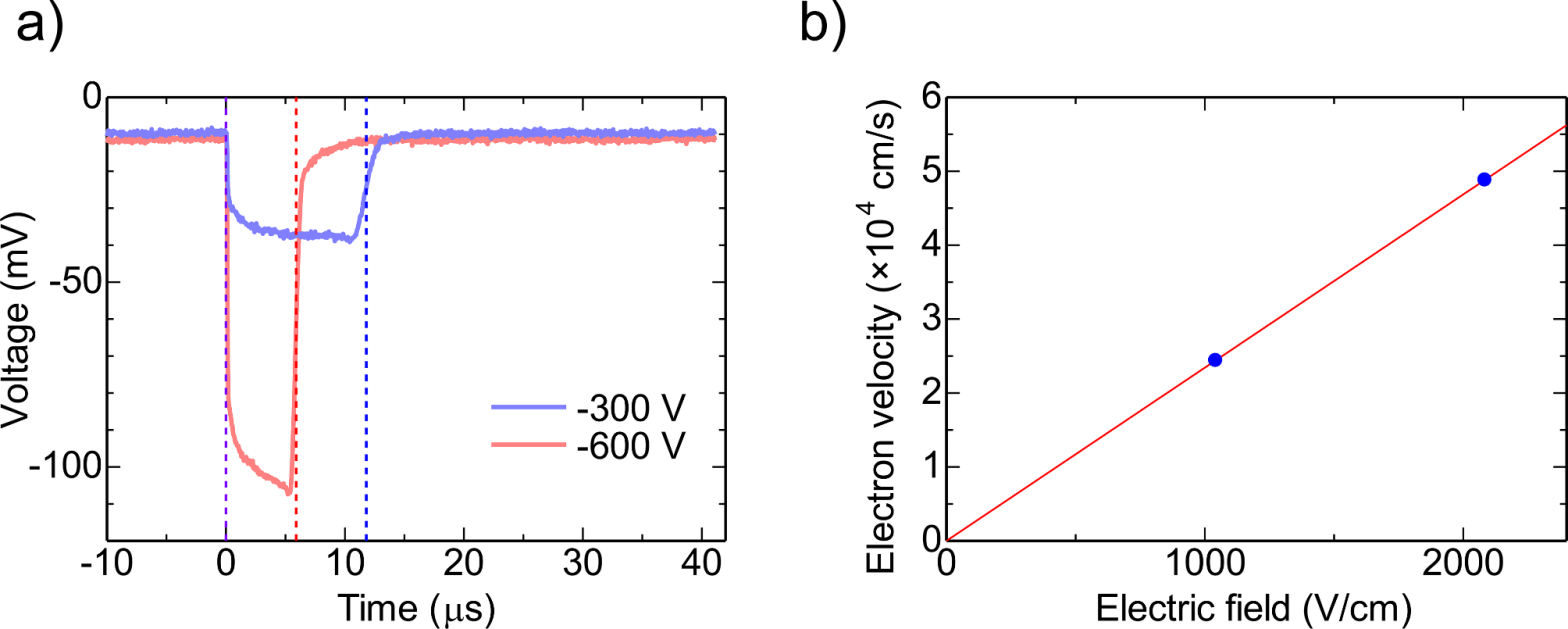
(**a**) Signal pulse shapes induced by the pulsed-laser irradiation and (**b**) electric field dependence of the carrier velocity. The signal was obtained at the center position of the upstream sample.

Figure 11a shows examples of the signal pulse shapes induced by the pulsed-laser irradiation. The pulsed laser irradiated the front electrode of the TlBr detector. The pulsed-laser irradiation produced a large number of electron-hole pairs just below the electrode that was irradiated by the pulsed laser. By applying a negative bias voltage to the front electrode, the holes were immediately collected at the front electrode. On the other hand, the electrons drifted to the back electrode by the applied electric field. During the movement of the electrons, a current signal was induced as shown in Fig. 11a. By measuring the time of flight or movement of generated electrons, the electron velocity can be determined. Of course, the electron velocity depends on the applied bias voltage. A relationship between the applied electric filed *E* and the electron velocity *v* is written by:

6 $$v=\mu E$$

where *µ* is the electron mobility. Figure 11b**)** shows the electric field dependence of the electron velocity. The electron mobility can be determined from the slope of this plot. The mobility was evaluated to be 23 cm^2^/Vs. This value is consistent with those obtained in previous studies^16,26^.

**Fig. 12:**
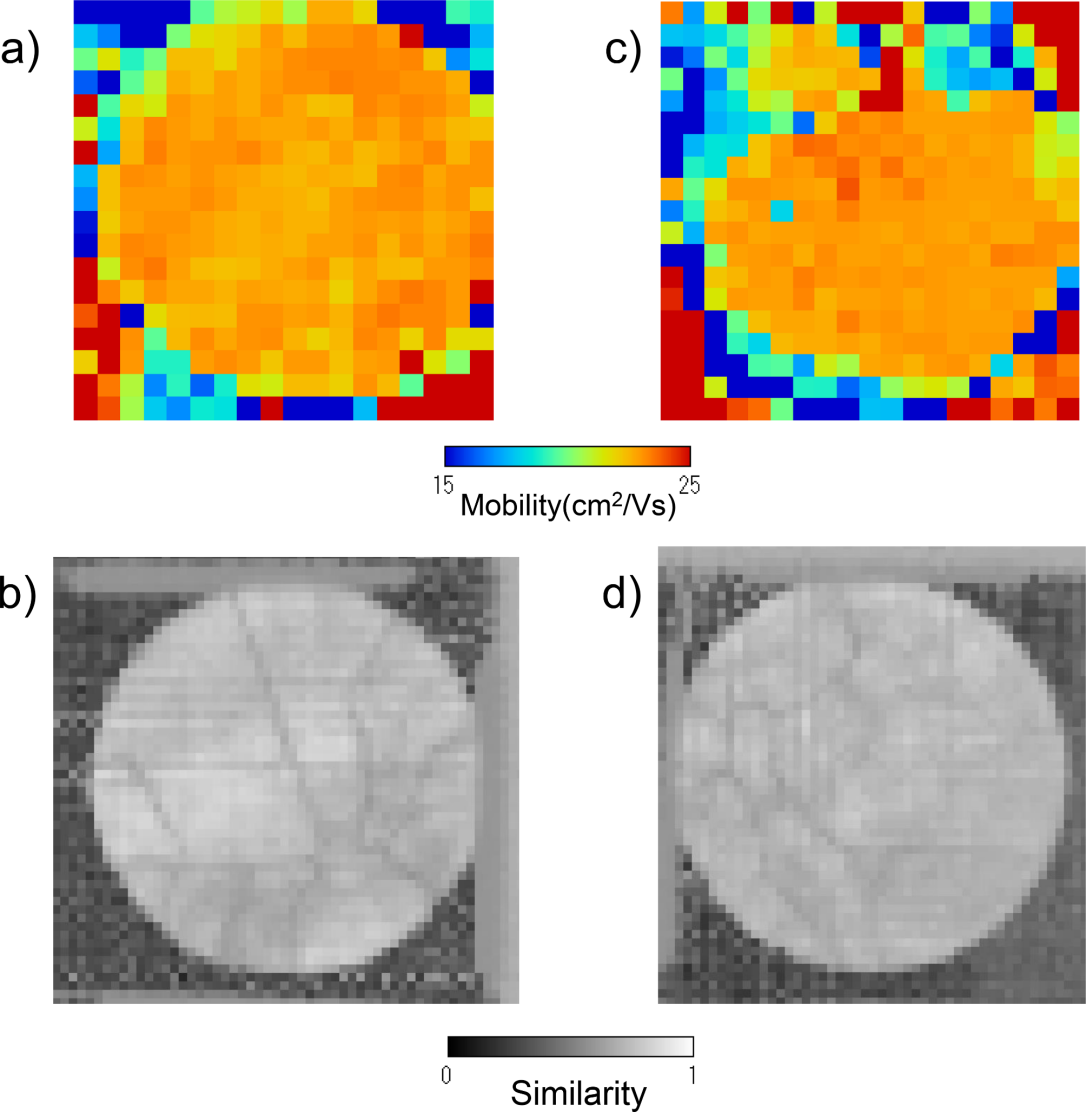
Electron mobility maps: (**a**,**b**) show the mobility for the upstream sample, while (**c**,**d**) display the mobility for the downstream sample. Maps (**a**,**c**) display the electron mobility maps obtained using the time-of-flight method for pulsed-laser-induced carriers. For comparison, the similarity maps obtained by neutron Bragg-dip analysis are displayed again in (**b**,**d**).

By scanning the TlBr detector sample, the electron mobility can be measured in two dimensions. Figure 12 shows the electron mobility maps obtained using the time-of-flight method for pulsed-laser-induced carriers. For comparison, the similarity maps obtained in the neutron Bragg-dip analysis are displayed again in Fig. 12b,d. The upstream sample shows an almost uniform distribution of electron mobility. On the other hand, the downstream sample exhibits regions with lower mobility in the upper part. Regions where the crystal orientations are not aligned and the similarity is low, are considered to have lower mobility. This is because such regions likely contain many defects and dislocations, which could serve as carrier trap sites. However, the experimental results do not show a correlation between the crystal alignment and the carrier mobility. Although the upstream sample contains some grain boundaries and regions with poor crystal alignment, it exhibits high carrier mobility across all areas. Although the downstream sample shows crystal quality similar to the upstream sample, it exhibits low carrier mobility in some areas. The downstream sample tends to contain more impurities due to the segregation of impurities during crystal growth. At least within the range of carrier mobility currently obtained, the effect of crystal integrity on carrier mobility is considered to be less significant than that of impurities. However, since there is still potential for significant enhancement in carrier mobility by achieving higher crystal integrity, it is also important to consider improving the crystal growth process. Additionally, while this study has considered only carrier mobility, the carrier lifetime has not been evaluated. To improve the most important detector performance factor, carrier collection efficiency, both carrier mobility and lifetime should be enhanced. A comparison between carrier lifetime and crystal quality is a task for future work.

## Conclusion

We investigated the crystal orientation distribution and carrier transport characteristics of TlBr semiconductor detectors using neutron Bragg-dip imaging and the time-of-flight method for pulsed-laser-induced carriers. Neutron Bragg-dip imaging has proven to be an effective method for determining the crystal orientation distribution of TlBr crystals. The results obtained from this method were consistent with those obtained from EBSD. While neutron Bragg-dip transmission can provide information from deeper regions of samples, EBSD is limited to analyzing only the surface. The crystal orientation maps obtained using neutron Bragg-dip imaging indicated imperfections in crystal growth, such as the presence of grain boundaries.

The time-of-flight measurements of pulsed-laser-induced carriers provided valuable information on the carrier transport characteristics in TlBr detectors. As a result of comparing the upstream and downstream samples, it was found that although both exhibited similar crystal quality, only the downstream sample had regions with low mobility. This suggests that the effect of crystal quality on carrier mobility is relatively small compared to other factors, such as concentrations of impurities. In this study, we have only considered carrier mobility and have not taken carrier lifetime into account. To improve carrier collection efficiency, both carrier mobility and lifetime should be improved. Therefore, it is still important to improve the crystal growth processes.

Finally, we conclude that the combination of neutron Bragg-dip imaging and time-of-flight carrier mobility measurements provides a comprehensive approach to evaluating and improving TlBr detectors. Future work should focus on further enhancing crystal growth techniques to minimize imperfections and improve carrier collection efficiency by evaluating both carrier mobility and lifetime.

## Data Availability

Data is provided within the manuscript files.
